# The Relationship Between Resilience and Posttraumatic Growth Among the Primary Caregivers of Children With Developmental Disabilities: The Mediating Role of Positive Coping Style and Self-Efficacy

**DOI:** 10.3389/fpsyg.2021.765530

**Published:** 2022-01-04

**Authors:** Wan Lu, Chen Xu, Xiankang Hu, Ju Liu, Qianhui Zhang, Li Peng, Min Li, Wenzao Li

**Affiliations:** ^1^Department of Military Psychology, Faculty of Medical Psychology, Army Medical University, Chongqing, China; ^2^Department of Cardiology, The First Affiliated Hospital of Chongqing Medical University, Chongqing, China; ^3^College of Basic Medical Sciences, Army Medical University, Chongqing, China; ^4^Department of Pediatrics, The Second Affiliated Hospital of Army Medical University, Chongqing, China

**Keywords:** posttraumatic growth (PTG), resilience, positive coping style, self-efficacy, primary caregivers, developmental disabilities

## Abstract

This study was conducted to investigate the relationship between posttraumatic growth (PTG), resilience, positive coping style, and self-efficacy among the primary caregivers of children with developmental disorders in Chongqing, China. A total of 198 primary caregivers (parents and grandparents) aged from 22 to 66 years old (*M* = 35.55, SD = 9.16), including 155 females (78.3%) and 43 males (21.7%), were enrolled. The Posttraumatic Growth Inventory, Connor-Davidson Resilience Scale-10, Simplified Coping Style Questionnaire, and General Self-Efficacy Scale were used for data collection. The results found that PTG could be positively predicted by resilience. Positive coping style and self-efficacy mediated the relationship between resilience and PTG. The different levels of PTG were determined by the resident location, monthly income and education of the primary caregivers. The results suggest that it is critical to improve the mental health of the primary caregivers (parents and grandparents) of children with developmental disabilities. Our results also provide a scientific basis for future research.

## Introduction

In China, approximately 4.04 million (6.65%) children are diagnosed with developmental disabilities (Olusanya et al., 2018). “Developmental disability” is an umbrella term that contains a group of conditions resulting from impairments that affect a child’s physical, learning, or behavioral functioning (Centers for Disease Control and Prevention (CDC), 2018, p.1). Affected children typically have cerebral palsy, autism spectrum disorder (ASD), attention deficit/hyperactivity disability (ADHD), intellectual disability, or other language/speech disabilities (American Psychiatric Association, 2013). In recent years, the impact of a diagnosis of developmental disabilities in children on primary caregivers has become better understood (Ribeiro et al., 2016). Caregiving for children who have been diagnosed with developmental disabilities leads adverse psychological health effects such as stress (Meadan et al., 2010; Zaidman-Zait et al., 2017), depression (Davis and Carter, 2008; Picardi et al., 2018), and anxiety (Al-Farsi et al., 2016; Sohmaran and Shorey, 2019) and has physical health impacts such as frequent challenges sleeping (Levin and Scher, 2016), eating (Bouma and Schweitzer, 1990) or communicating and behavioral and health complications (Ying et al., 2021). In addition, when caring for a child with developmental disabilities, caregivers can also experience posttraumatic stress symptoms (PTSSs), such as intrusive recollections, avoidant symptoms and hyperarousal symptoms (Wijesinghe et al., 2015; Masefield et al., 2020). Most studies have focused on the burdens (Alnazly and Abojedi, 2019; Hong et al., 2020; Liao and Li, 2020). Recently, under the influence of positive psychology, researchers have shifted their research focus to positive changes in individuals, such as posttraumatic growth (Hefferon et al., 2009), resilience (Aburn et al., 2016), positive coping style and self-efficacy (Luque Salas et al., 2017) and other similar concepts.

### Posttraumatic Growth

Although the term “trauma” is used in the stress and coping literature to describe events such as death and disaster, it is also used to describe a subjective response to an event that overwhelms an individual’s emotional, cognitive, or physical ability to cope (van der Merwe and Hunt, 2019). Meanwhile, evidence shows that subjective experience and supportive growth that promotes personal development (Scorgie and Sobsey, 2000; Blacher and Baker, 2007; Alon, 2019) will occur to the primary caregivers of a child with autism after facing difficulties (Waizbard-Bartov et al., 2019). Posttraumatic growth (PTG) (Tedeschi and Calhoun, 2004) is defined as the process of creating meaning following a stressful situation, which can be expressed in positive ways, including feelings of strength and power, improvement in interpersonal relations, spiritual development, and finding meaning in life (Linley and Joseph, 2004; Taubman-Ben-Ari et al., 2010). The term is generally applied to situations that have far-reaching impacts on various aspects of life (Alon, 2019). Evidence has shown that caregivers of children with disabilities demonstrate considerable strength and spiritual and personal growth, all of which bear similarities to posttraumatic growth (Phelps et al., 2009). In other words, a child’s disability may also make a positive contribution to the lives and well-being of caregivers (King et al., 2006; Bayat, 2007; Benson, 2010).

### Resilience

Resilience is a protective factor for PTG elevation (Qin et al., 2021). As previously mentioned, families of children with developmental disabilities confront special stressors, such as communication barriers and unpredictable behavior, so they are more susceptible to greater pressure (Wayment et al., 2019) and more complex challenges (Wayment and Brookshire, 2018) than families of typically developing children. Despite such enormous challenges, caregivers still have high expectations for their families and often manage to maintain a sense of control with active adaptation and response (Lickenbrock et al., 2011), namely, resilience (Kapp and Brown, 2011). Resilience can promote the production of PTG among the primary caregivers of children with autism, as high resilience contributes to social support (Hastings and Brown, 2002), optimism (Maercker and Zoellner, 2004), personal strength (Kapp and Brown, 2011; Zhang et al., 2015), and other factors that enable primary caregivers to better deal with life’s stressful events. Resilience can shift the individual perspective from problem to advantage and enhance the individual’s positive experiences.

### Positive Coping Style

The development of PTG is also closely related to positive coping styles (Manor-Binyamini, 2012), including adaptive and active coping processes. It enables caregivers to find meaning in difficult situations, promotes their psychological growth, and affects both the level of experienced stress and resilience (Shepherd et al., 2018). As Gray (1994) suggests there is no single coping style that could be universally applied to reducing the pressure parents face; the key is to adopt proper coping styles (Lambert and Lazarus, 1970).

### Self-Efficacy

Posttraumatic growth not only relieves caregivers’ anxiety and depression but also increases their self-efficacy and confidence in the face of the children’s disorders and their recovery. Studies (Kuhn and Carter, 2006; Zhang et al., 2015) have shown that improvements in caregivers’ self-efficacy occur when their children give them positive feelings and confidence in caregiving. It helps them depreciate their “past self” to maintain a favorable view of their “present self” (Zhang et al., 2015, p.6). Self-efficacy gives caregivers a greater sense of control and confidence in their roles as parents, teachers, and nurses and affects their cognitive reframing (Kuhn and Carter, 2006). A study on 19 Israeli parents of children with autism showed that the improvement in self-efficacy, namely, emotional coping abilities, could promote the generation of PTG in mothers (Waizbard-Bartov et al., 2019).

### Research Aim

Most related research has been limited to mothers of children with autism spectrum disorders, but few studies have acknowledged that the resilience, positive coping style and self-efficacy of the primary caregivers of children with developmental disabilities affect the production of PTG in caregivers. Even less is known about the underlying mechanism by which these factors influence PTG. One study (Wu et al., 2021) has shown that positive coping style and resilience mediate the relationship between perceived social support and PTG among primary caregivers of schizophrenic patients. Hence, based on previous research, this study aims to explore the relationships among the positive coping style, self-efficacy and PTG of such caregivers and to analyze the mediating role of positive coping style and self-efficacy in the relationship between resilience and PTG and whether primary caregivers of children with developmental disabilities experience PTG.

## Materials and Methods

### Participants and Procedures

Due to the particularity of the research participants, the sample size was calculated as follows: The scale (Post-Traumatic Growth Inventory, PTGI) had the largest number of items among the four scales. The sample size was 5–10 times the number of items (21) in this scale (21*5 = 105∼21*10 = 210), i.e., 105∼210 samples. In this study, a convenience sample of 200 participants was recruited from the Children’s Rehabilitation Center of the Pediatric Department of the Second Affiliated Hospital of the Army Medical University and DanFu Children’s Rehabilitation Institute. In practice, the sample size that signed informed consent and could be included in our study according to the inclusion criteria was relatively small. The researchers ended up with a total sample size of 200 at the two hospitals. The participants were the primary caregivers of children with diagnosed developmental disabilities, including parents and grandparents. The inclusion criteria were as follows: (1) being the primary caregiver of a child with developmental disabilities; (2) having children diagnosed with developmental disabilities by a pediatrician; (3) having basic reading comprehension ability and no communication barriers; (4) serving as a daily caregiving for at least 8 h; and (5) signing informed consent forms. The exclusion criteria were as follows: (1) serving as a caregiver for no more than 6 months within one periodic year and (2) having a mental disorder.

A cross-sectional survey method was adopted to conduct a field questionnaire survey on the included subjects during September and November 2020. A total of 200 questionnaires were issued and collected, of which 198 were valid (two questionnaires were not completed), for a valid rate of 99%. There were 43 (21.7%) males and 155 (78.3%) females aged 22 to 66 years (*M* = 35.55; SD = 9.16). Before participating in the survey, the caregivers were asked to provide consent and complete privacy policy forms to declare their voluntary participation, and they were free to decline to participate at any step of the survey. The participants were informed that their responses would be confidential. The purpose, content and procedures of the survey were explained in detail. The primary caregivers completed the questionnaire by themselves with the guidance of professionally trained investigators. Questionnaires were distributed and collected on the same day, after no more than 8 h.

### Measures

#### Posttraumatic Growth Inventory

The Chinese version of the Posttraumatic Growth Inventory (PTGI) has been shown to be a valid measure with excellent reliability and validity (Liu et al., 2014). The PTGI consists of 21 items divided into five dimensions: new possibilities, relating to others, appreciation of life, spiritual change, and personal strength. The measure uses a 6-point Likert scale ranging from 0 (never) to 5 (always). A higher score represents a higher level of posttraumatic growth. Cronbach’s alpha was 0.833 for the total score on this scale.

#### Connor-Davidson Resilience Scale-10

The Connor-Davidson Resilience Scale (CD-RISC-10; Connor and Davidson, 2003) is a 25-item scale assessing the mental resilience of individuals jointly compiled by Connor and Davison. In this sample, a 10-item simple Chinese version was adopted (Campbell-Sills and Stein, 2007; Wang et al., 2010). The 5-point Likert scale ranges from 0 (never) to 4 (always). The total score is calculated to reflect one’s resilience, with higher scores indicating greater resilience. In this study, the Cronbach’s alpha for this scale was 0.775.

#### Simplified Coping Style Questionnaire

The Chinese version of the Simple Coping Style Scale has good reliability and validity (Xie, 1998). It is composed of the two dimensions (subscale) of positive coping style and negative coping style, with 20 total items. The 4-point Likert scale ranges from 0 (never) to 3 (always). Cronbach’s alpha was 0.770 for the positive coping subscale and 0.651 for the passive coping subscale.

#### General Self-Efficacy Scale

The General Self-Efficacy Scale (GSES) is used to assess the overall self-efficacy of adults. The Chinese version adopted in this study has good reliability and validity (Wang et al., 2001). The scale uses a 4-point Likert scale ranging from 1 (strongly disapprove) to 4 (strongly approve). Item responses were summed and divided by 10 to form a scale score, with higher scores indicating a higher level of self-efficacy. The Cronbach’s alpha for this scale was 0.856.

#### Covariates

The covariates of this study include sociodemographic variables of the participants, namely, gender, age (a continuous variable), education level, monthly income (yuan per month), residence, marital status, and child’s diagnosis.

### Data Analysis

#### Preliminary Analyses

EpiData3.3 was used for data entry, and the statistical software SPSS-25 was used to perform statistical analyses. Prior to the test of the mediation model, preliminary analyses were conducted. First, the basic data of primary caregivers and children were analyzed by general statistics and ANOVA to test whether the sociodemographic characteristic data were significantly different. Second, Pearson correlations were run to examine the relationships among resilience, positive coping style, self-efficacy and PTG.

#### Testing the Proposed Model

**FIGURE 1:**
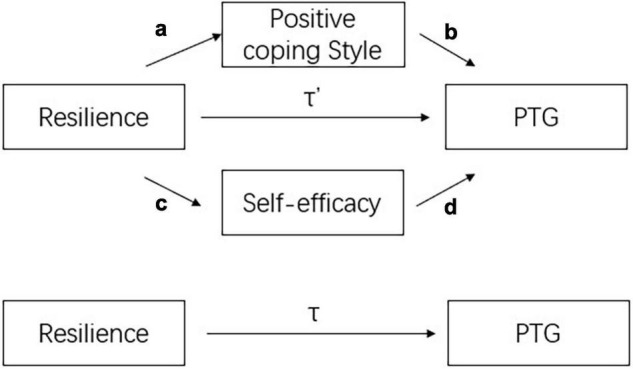
The conceptual framework of mediation model. τ is the unstandardized total effect of resilience on PTG; a is the unstandardized regression coefficient of resilience on positive coping style; b represents the unstandardized partial regression coefficient of positive coping style on PTG after controlling for the effect of resilience on PTG; c is the unstandardized regression coefficient of resilience on self-efficacy; d represents the unstandardized partial regression coefficient of self-efficacy on PTG after controlling the effect of resilience on PTG; and τ′ is the unstandardized direct effect of resilience on PTG.

The mediation tests were examined *via* the PROCESS macro in SPSS. Figure 1 displays the conceptual framework of the tested mediation model. The coefficient τ′ is a direct effect of resilience on PTG. Resilience might also indirectly influence PTG through a positive coping style and self-efficacy, and these effects are captured by the production of coefficients a, b, c, and d (i.e., ab, cd). Coefficient τ is the total effect of resilience on PTG, given no mediators. All coefficients (τ, τ′, a, b, c, d) are unstandardized.

The alpha level is 0.05 and helps determine whether τ′ and τ are statistically significant. The significance of the indirect effect was decided based on its 95% confidence interval (CI) estimated using the percentile bootstrap method. If the CI does not include zero, the corresponding effect is statistically significant.

## Results

### Preliminary Analysis

The preliminary analyses showed satisfactory asymmetry and kurtosis values for all items and a low missing rate (<1%). Researchers (Curran and Finch, 1996) recommend a score set with its skewness within the range of (−2, +2) and kurtosis within the range of (−7, +7) to indicate an approximately normal distribution (Curran and Finch, 1996). Accordingly, all score distributions in the current study were considered to follow a normal distribution (Table 1). Single factor analysis of variance showed significant education differences in PTG (*F*_(3,194)_ = 7.055, *P* < 0.001), significant income differences in PTG (*F*_(3,194)_ = 2.738, *P* = 0.045), and significant residence differences in PTG (*F*_(2,195)_ = 3.726, *P* = 0.026). However, the results also showed non-significant child’s diagnosis differences in PTG (*F*_(4,193)_ = 0.021, *P* = 0.999). Moreover, the *t*-tests showed non-significant marital status differences in PTG (*t*_(196)_ = −1.778, *P* = 0.770) (Table 2).

**TABLE 1:** Means (M), standard deviations (SD), kurtosis (KU), skewness (SK), correlations, and reliabilities.

Variables	M	SD	KU	SK	α	1	2	3
								
(1) Resilience	23.78	5.18	0.309	0.199	0.775	–		
(2) Positive coping style	1.92	0.44	−0.132	−0.037	0.770	0.448***	–	
(3) Self-efficacy	2.45	0.48	0.631	0.372	0.856	0.416***	0.429***	–
(4) PTG	61.99	12.04	0.550	−0.293	0.833	0.445***	0.595***	0.482***

α = Cronbach’s alpha, ***p < 0.001.

**TABLE 2:** Relationship between the social-demographic characteristic and the PTG.

Items		*n*	P(%)	PTG score	t\F	*P*
Education level	Junior school and below	52	26.2	59.44 ± 12.78	7.055(F)	0.000
	High school	31	15.7	55.65 ± 10.38		
	Junior college	63	31.8	63.44 ± 11.91		
	Bachelor degree and above	52	26.3	66.58 ± 10.30		
Monthly income (yuan per month)	<3,000	23	11.6	57.35 ± 12.67	2.738(F)	0.045
	3,000–5,000	39	19.7	59.15 ± 12.94		
	5,000–8,000	62	31.3	63.71 ± 12.10		
	>8,000	74	37.4	63.50 ± 10.84		
Residence	Urban area	111	56.0	63.57 ± 11.15	3.726(F)	0.026
	Suburb	54	27.3	61.72 ± 11.57		
	Rural area	33	16.7	57.15 ± 14.53		
Marital status	Married	190	96.0	61.68 ± 11.87	−1.778(t)	0.770
	Divorced	8	4.0	69.38 ± 14.60		
Diagnosis	ADHD	19	9.6	61.95 ± 9.61	0.021(F)	0.999
	Intellectual developmental disabilities	30	15.2	62.53 ± 10.55		
	Speech/language developmental disabilities	48	24.2	61.85 ± 10.93		
	Cerebral palsy	42	21.2	62.07 ± 13.89		
	ASD	59	29.8	61.80 ± 13.23		

*n = sample size. P(%) = percentage of total sample. Diagnosis = child’s diagnosis. PTG score = PTGI scale score(M ± SD). t = the t-value in the independent sample t-test. F = the F-value in the one-way ANOVA. P = P-value.*

### Correlations and Reliabilities

We performed an internal consistency reliability analysis using Cronbach’s alpha (α). According to the results, the α reliability of the resilience scale was 0.775 (*M* = 23.78, SD = 5.18), 0.770 (*M* = 1.92, SD = 0.44) for the positive coping style scale, 0.856 for the self-efficacy scale (*M* = 2.45, SD = 0.48), and 0.833 for the PTG scale (*M* = 61.99, SD = 12.04). These results indicate that the reliability of all scales used in the study was adequate. The correlation coefficients showed moderate relationships between the variables. PTG had a positive correlation with resilience (*r* = 0.445, *P* < 0.001), positive coping style (*r* = 0.595, *P* < 0.001) and self-efficacy (*r* = 0.482, *P* < 0.001). In addition, self-efficacy had a positive correlation with resilience (*r* = 0.416, *P* < 0.001) and a positive coping style (*r* = 0.429, *P* < 0.001). There was also a positive correlation between resilience and positive coping style (*r* = 0.448, *P* < 0.001). The means, standard deviations, α values, and correlations between factors are presented in Table 1.

### Mediating Effect of Positive Coping Style and Self-Efficacy on the Relationship Between Resilience and Posttraumatic Growth

Prior to the conditional process analysis, we controlled for potential multicollinearity issues between the variables *via* collinearity statistics. In the collinearity analysis, the variance inflation factor (VIF) ranged from 1.33 to 1.37, as the VIF values were less than 3, indicating no danger of multicollinearity (Hair et al., 2010). To analyze the mediating effect of positive coping style and self-efficacy, we used Model 4 in Hayes (2017) the macro program PROCESS. The mediation effect was tested after controlling for gender, age, education level, monthly income (yuan per month), residence, marital status, and child’s diagnosis.

The results showed that the total effect of resilience on PTG was significant (τ = 0.904, *P* < 0.001), and after the mediation variables of positive coping style and self-efficacy were put into the model, the direct effect was statistically non-significant (τ′ = 0.291, *P* = 0.075). In addition, resilience had a significant predictive effect on positive coping style (*a* = 0.035, *P* < 0.001), and the predictive effect of positive coping style on PTG was also significant (*b* = 11.415, *P* < 0.001). Resilience had a significant predictive effect on self-efficacy (*c* = 0.037, *P* < 0.001), and the predictive effect of self-efficacy on PTG was also significant (*d* = 5.678, *P* < 0.001) (Table 3). We further generated 5,000 bootstrapping samples from the original dataset by random sampling to assess the size of the indirect effect. The results indicated that this indirect effect was ab + cd = 0.613, 95% CI = [0.363, 0.885] (ab = 0.402, 95% CI = [0.204, 0.623] and cd = 0.211, 95% CI = [0.075, 0.380]). The empirical 95% CI did not include zero, indicating that positive coping style and self-efficacy were paths through which resilience could influence PTG. Among them, the direct effect was 0.291, and the mediation effect was 0.613, accounting for 32 and 68% of the total effect, respectively (Table 3). Altogether, the model accounted for 44.9% of the total variance in PTG.

**TABLE 3:** Mediation effect model.

Predictors	M1 (PTG)				M2 (Positive coping style)				M3 (Self-efficacy)				M4 (PTG)			
	B	*t*	LLUI	ULCI	B	*t*	LLCI	ULCI	B	*t*	LLCI	ULCI	B	*t*	LLCI	ULCI
Gender	−1.583	−0.837	−5.312	2.145	−0.751	−1.099	−0.209	0.596	−0.157	−2.083*	−0.307	−0.008	0.170	0.103	−3.056	3.396
Age	−0.008	−0.089	−0.184	0.168	−0.006	−1.815	−0.123	0.001	−0.001	0.125	−0.006	0.007	0.056	0.730	−0.096	0.209
Education	1.644	1.979*	0.006	3.283	0.049	1.661	−0.094	0.109	0.021	0.649	−0.044	0.087	0.952	1.332	−0.458	2.364
Income	−0.585	−0.647	−2.370	1.198	−0.123	−0.377	−0.768	0.522	−0.060	−1.659	−0.131	0.011	−0.103	−0.133	−1.640	1.432
Residence	−1.371	−1.225	−3.577	0.835	−0.027	−0.690	−0.107	0.052	−0.045	−1.022	−0.134	0.426	−0.792	−0.826	−2.684	1.099
Marital status	5.745	1.422	−2.221	13.712	0.091	0.625	−0.196	0.379	0.364	2.252*	0.045	0.683	2.633	0.752	−4.267	9.535
Diagnosis	0.090	0.155	−1.062	1.244	0.034	1.616	−0.007	0.075	0.001	0.048	−0.045	0.047	−0.305	−0.607	−1.299	0.687
Resilience	0.904	5.313***	0.568	1.240	0.035	5.728***	0.023	0.047	0.037	5.449***	0.023	0.050	0.291	1.786	−0.030	0.613
Positive Coping Style													11.415	6.341***	7.864	14.966
Self-efficacy													5.678	3.497***	2.475	8.881
R^2^	0.238				0.248				0.233				0.449			
F	7.378***				7.828***				7.175***				15.246***			
Indirect effect									B		Boot SE		LLCI		ULCI	
Positive coping style									0.402		0.108		0.204		0.623	
Self-efficacy									0.211		0.079		0.075		0.380	

Gender, male = 1. Age is coded as a continuous variable. Education: Under middle school = 1. Income: Under 3,000 = 1. Residence: Urban area = 1. Marital status: Married = 1. Diagnosis: ADHD = 1. LL = low limit, CI = confidence interval, UL = upper limit. *p < 0.05, ***p < 0.001.

## Discussion

Our goal in this study was to extend our understanding of the relationship of resilience, positive coping style, self-efficacy and PTG among the primary caregivers of children with developmental disabilities. Specifically, we hypothesized that resilience directly or indirectly predicted the generation of PTG among primary caregivers through positive coping styles and self-efficacy. Our results confirmed the hypothesis: (1) higher resilience was associated with higher levels of PTG, consistent with previous findings (Kalisch et al., 2015; Qin et al., 2021), and the primary caregivers with higher resilience were more likely to actively seek multiple ways of solving problems, to take the initiative to obtain relevant knowledge and more easily accept their reality, to face their children with optimism, and to try to return to a normal life (Owens, 2016); (2) for the mediating role of self-efficacy, primary caregivers with higher resilience acquired a sense of achievement when handling pressure-related problems, which enhanced their sense of self–efficacy and thus promoted more PTG; and (3) positive coping style played a stronger mediating role in the relationship between resilience and PTG. The higher the resilience of caregivers was, the more active they were in exploring solutions to problems, calmly coping with stressful events in life, and improving their psychological growth, consistent with previous research results (Kuhn and Carter, 2006; Stephenson et al., 2016). Moreover, resilience also plays a key role when caregivers are confronted with adverse events in their lives. For instance, they will actively seek help, such as asking family members and friends for solutions, searching about relevant issues on the internet, and supporting their “companions” who have similar experiences. These actions can lead them to achieve a certain inner growth, hence promoting more PTG behaviors.

Consistent with previous findings (Qin et al., 2021), our study revealed that the primary caregivers of children with developmental disabilities experienced moderate PTG. We also found statistically significant differences in PTG across the education levels of the primary caregivers. According to the results of our study, subjects with a college education or higher (accounting for 58.1% of the sample) generally had higher PTG scores than subjects with a lower educational level. As we assume, perspective and the way in which life events are processed vary with level of education, hence affecting PTG (Stockton et al., 2011; Magallón-Neri et al., 2017). The different levels of PTG resulting from both area of residence and monthly income, which are rarely mentioned in previous articles, have also been shown in this study. There was a progressive decrease in PTG scores from urban areas to rural areas. This variation may have been caused by the differences in living conditions (economic level), educational concepts and social support in various areas of residence, concerning both caregivers and children. For instance, the socioeconomic status of people who live in rural areas may be lower than that of people who live in urban areas. Low-income families may be under greater pressure to pay for their children’s rehabilitation, which negatively affects PTG. Moreover, in traditional Chinese culture, disability is viewed as a punishment for the disabled person’s sins in the past life or the sins of the person’s parents (Huang et al., 2009; Holroyd, 2016), so primary caregivers consider the disabilities to be stigmatic. Furthermore, social support for caregivers may also be insufficient in rural areas, where more negative public attitudes could lead to lower expectations and more discriminatory behaviors and marginalization of families with disabilities than in the suburbs and urban areas (Zheng et al., 2016). Our study also found non-significant marital status and child diagnosis differences in PTG. PTG did not differ significantly in dependence on the marital status, mainly because there were only 8 (4%) divorced primary caregivers in our sample size, which accounted for a small part of the sample size. PTG did not differ in dependence on the child’s diagnosis. We suspect that developmental disabilities, regardless of the diagnosis, pose a great challenge for the primary caregiver. For example, developmental disabilities need rehabilitation treatment, which is a long process that requires considerable time, energy and money (Blachera et al., 2005; Wayment et al., 2019). Therefore, targeted psychological intervention is particularly important for caregivers.

## Implications for Practice

The families of children with developmental disabilities face great economic, life, and other pressures. Thus, the primary caregivers of children with developmental disabilities might have significant psychological problems, gradually resulting in a public health problem. Most previous studies are limited to the exploration of PTG and the adverse factors (Alnazly and Abojedi, 2019; Bravo-Benítez et al., 2019; Naheed et al., 2020) and protective factors (Blackledge and Hayes, 2006; Young and Kleist, 2010; Wayment and Bauer, 2018) of PTG in the parents of ASD children. This study included the primary caregivers of ASD children and children with other developmental disabilities, such as ADHD, intellectual disability and other developmental delays. While exploring the protective factors of the primary caregivers of children with developmental disabilities, we found that resilience directly and indirectly affected the production of PTG through positive coping styles and self-efficacy, and that some sociodemographic characteristics significantly affected PTG. The results also improve our understanding of PTG among the primary caregivers of children with developmental disabilities.

The mental health of caregivers will affect the future treatment and rehabilitation of their children, so it is critical to provide psychological intervention on primary caregivers (Sohmaran and Shorey, 2019). In addition, an interesting finding of our study is that some of the preschool children’s grandparents became their primary caregivers, which should be closely related to China’s unique cultural background. However, there are only very few studies (Hillman and Anderson, 2019) abroad that examined the positive psychology of the grandparents of children with developmental disabilities, and no such studies are found in China for the time being. This paper may provide a new idea: It may be helpful to encourage mental health personnel to provide substantial care not only to parents but also to grandparents when focusing on the mental health of the primary caregivers of children with developmental disabilities. Certain psychological interventions may be needed for grandparents to improve the psychological health of the whole family. While focusing on the improvement in PTG, psychological workers should also pay attention to the assessment and intervention of resilience, coping style and self-efficacy level, and the subsequent influence on PTG, to more effectively help the families deal with pressure, seek professional help, and improve family and children’s life quality and treatment. Finally, the progress of children with developmental disabilities requires the joint efforts of medical staff and primary caregivers, so it is necessary for the staff of medical rehabilitation institutions and special education institutions to focus on the primary caregivers of children with developmental disabilities.

## Limitations and Future Directions

The results of this study should be considered in light of the following three limitations. First, as the study relies on cross-sectional self-reported data with a relatively small sample size, it does not allow the identification of causality or generalization. Second, this research is based on a field survey conducted in two children’s rehabilitation institutions in the city of Chongqing; therefore, caregivers who did not apply for medical care or treatment in the institutions were excluded due to possible negligence or unawareness of the children’s situation and economic deprivation of the family, which could lead to sampling bias. Third, self-reported measures were used to assess resilience, positive coping style, self-efficacy and PTG; therefore, the results could have been impacted by common method variance. Future studies should include more samples of grandparents among primary caregivers and independently explore their PTG and the differences between grandparents and parents in detail, adding the third-person estimations apart from the self-reports so data have a meaning to the clinical practitioner. Moreover, this population can be followed longitudinally to examine causality. In addition, personality traits (optimism, self-awareness, and sense of responsibility, etc.) were not included in this study and should be explored in more dimensions in future studies.

## Conclusion

Our study showed that the primary caregivers of children with developmental disabilities experienced PTG, while the education background, monthly income and residence of caregivers also had a significant impact on their PTG. Additionally, resilience directly and indirectly had a positive predictive effect on the PTG of the primary caregivers of children with developmental disabilities through a positive coping style and self-efficacy. This study further confirmed the important role of resilience in PTG generation. It enables primary caregivers to positively deal with stress and difficulties and increase their self-efficacy. Our research provides a scientific basis for the psychological and behavioral intervention of primary caregivers by clinical psychology and social workers, which can help primary caregivers to better cope with stress and traumatic events, and promote the emergence of PTG, which may be very important for their mental health (Gori et al., 2021).

## Data Availability Statement

The original contributions presented in the study are included in the article/supplementary material, further inquiries can be directed to the corresponding authors.

## Ethics Statement

The studies involving human participants were reviewed and approved by the Medical Ethics Committee of Army Medical University, PLA (2020 NO.035-03). The patients/participants provided their written informed consent to participate in this study.

## Author Contributions

WL contributed to the experiment design, did the experiment, analyzed the data, and contributed to article writing and editing. CX did the experiment and data analysis. XH did the experiment. JL and QZ contributed to article language revision. LP edited the article. ML contributed to the experimental design, supervision of the experiment, and project funding acquisition. WZL dealt with unexpected situations and experiment supervision in the study. All authors contributed to the article and approved the submitted version.

## Conflict of Interest

The authors declare that the research was conducted in the absence of any commercial or financial relationships that could be construed as a potential conflict of interest.

## Publisher’s Note

All claims expressed in this article are solely those of the authors and do not necessarily represent those of their affiliated organizations, or those of the publisher, the editors and the reviewers. Any product that may be evaluated in this article, or claim that may be made by its manufacturer, is not guaranteed or endorsed by the publisher.
